# A severely encrusted forgotten double - J ureteral catheter with giant stone formation

**DOI:** 10.1590/S1677-5538.IBJU.2018.0330

**Published:** 2019

**Authors:** Gaurav Garg, Deepanshu Sharma, Siddharth Pandey, Manoj Kumar

**Affiliations:** 1 King George's Medical University, Lucknow, India

## CASE PRESENTATION

**Figure 1:**
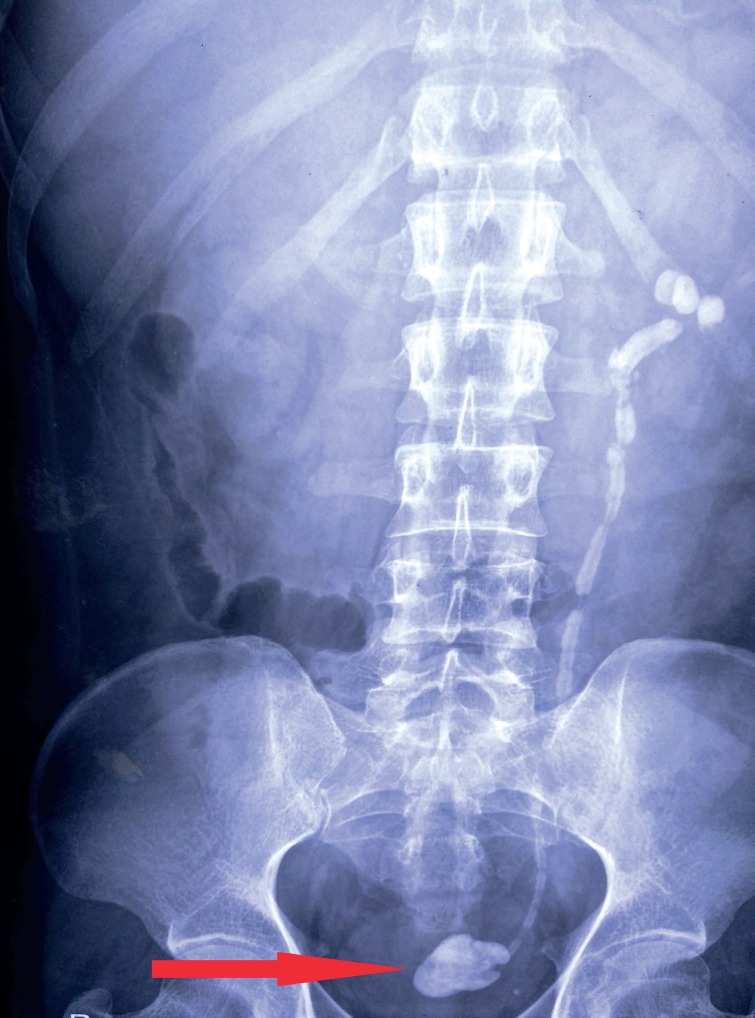
X ray KUB image depicting completely encrusted double - J ureteral catheter with giant stone formation at distal bladder end resembling a “hockey - stick”(red arrow).

**Figure 2:**
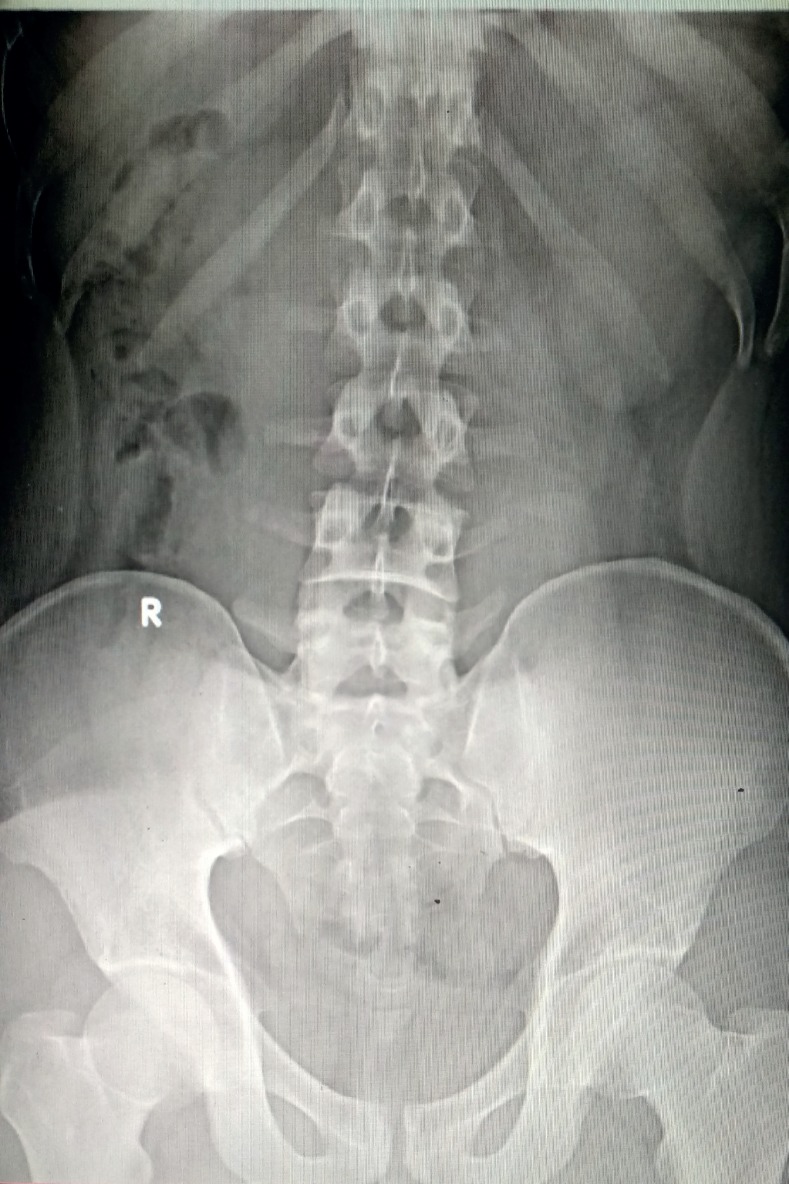
X ray KUB image depicted no evidence of radio - opaque shadow at 3 - months follow-up.

A 45 - year - old male patient presented with pain in left flank and voiding lower urinary tract symptoms (LUTS) for the last 12 months. He also presented history of several episodes of urinary tract infections (UTI) in the last 2 years. There was a non - documented history of undergoing left open pyelolithotomy for left renal calculus 5 - years ago. Apart from a scar present in left flank region, the rest of the general physical examination was unremarkable. Routine blood / urine examination revealed normal haemoglobin, blood counts, kidney function tests, random blood sugar levels and presence of 20 - 25 pus cells / hpf. Urine culture examination revealed presence of E coli (> 10^5^ CFU / ml). Imaging with X - ray and ultrasound of KUB region demonstrated completely encrusted double - J ureteral catheter with giant stone formation (45 mm) at distal bladder end resembling a “hockey - stick” (Figure-1). After proper counselling and consent, the patient underwent removal of encrusted ureteral catheter by combined endourological approaches - cystolithotripsy (CLT), retrograde ureteroscopic lithotripsy (URSL) and percutaneous nephrolithotripsy (PCNL) performed in two sessions. Post - operative period was uneventful. At 3 - months follow-up there is no evidence of any radio - opaque shadow in X Ray KUB (Figure-2).

## DISCUSSION

Double - J ureteric catheters are an indispensable part of urology. Close follow-up of these patients is mandatory to prevent long - term complications arising from prolonged in situ ureteral catheter such as worsening of hydronephrosis, infections, encrustations, stent migration and renal damage (1). Combined use of endourological approaches (CLT, URSL, PCNL etc.) may be required for successful management of forgotten and severely encrusted ureteral catheter (2). X ray KUB along with non - contrast computerized tomography (NCCT) can act as valuable imaging modalities for evaluation of encrusted and forgotten ureteral catheters. The best way to minimise the burden of encrusted and forgotten ureteral catheters is to prevent them by developing a strong recall system. The data pertaining to patient's name, address, contact number / e-mail id, expected date of ureteral catheter removal must be stored within the hospital. Patient and their relatives must be thoroughly counselled regarding the need, expected complications and timely removal of ureteral catheters. Use of a computerized warning and stent retrieval system can be made for the defaulters (3).
